# Investigation into the psychological impact of the COVID-19 pandemic for people living with HIV

**DOI:** 10.1177/09564624231179275

**Published:** 2023-06-03

**Authors:** Sze Wing Karina Lo, Luke Muschialli, Thomas Fernandez, Colette Smith, Dimitra Peppa, Fiona Burns

**Affiliations:** 1 University College London, 4919 Division of Infection and Immunity, London UK; 2 4965 Royal Free London NHS Foundation Trust, London, UK; 3 Institute for Global Health, University College London, London, UK; 4 Mortimer Market Centre, Department of HIV, CNWL NHS Trust, London UK; 5 HIV Medicine, Royal Free London NHS Foundation Trust, London UK

**Keywords:** Epidemiology, Europe, HIV (human immunodeficiency virus), location, other, viral disease

## Abstract

**Background:**

People living with HIV (PLWH) report high levels of anxiety. This study assessed the prevalence of COVID-19-related anxiety in PLWH.

**Methods:**

Participants were recruited from two UK HIV clinics (01/03/2020 - 30/05/2022) and asked to complete the Coronavirus Anxiety Scale. The proportion with scores ≥9 (cut-off for dysfunctional pandemic-related anxiety) and ≥1 (reporting of *any* pandemic-related anxiety) were analysed.

**Results:**

115 PLWH were included, predominantly identifying as male (83.5%, *n* = 96), white (58.3%, *n* = 67) and reporting post-secondary education (82.6%, *n* = 95), with a median age of 51 years (range 22–93). Median CAS score was 0, with 4.4% scoring ≥9 (*n* = 5). More women scored ≥9 than men (16.7% (*n* = 3) and 2.1% (*n* = 2) respectively). Black African (13.6%, *n* = 3) and Other Ethnic Minority PLWH (25%, *n* = 2) had a greater proportion of scores ≥9 than White/Asian PLWH (both 0%). SARS-CoV-2 exposure was associated with scores greater than 1 but not greater than 9. CAS score was not associated with lower CD4 (<350 cells/mm^3^), detectable HIV viral load (≥50 copies/ml), or a history of pre-pandemic anxiety.

**Conclusions:**

Pandemic-related anxiety was low, but we identified a sub-population reporting dysfunctional pandemic related anxiety. Future work should further investigate the psychological impact of the pandemic on this group.

## Introduction

The COVID-19 pandemic disrupted many aspects of life, and the measures brought in to limit the spread of SARS-CoV-2 had notable psychological impacts on the general population.^1^ In the UK, people living with HIV (PLWH) were identified as extremely clinically vulnerable, and advised to limit contact outside of their households,^2^ potentially exacerbating the mental health impact of the pandemic on this population. The detrimental impact on the mental health of PLWH during the pandemic has been reported in multiple settings.^3–10^ There are many potential drivers of this. From a health perspective, PLWH have voiced concerns about being at greater risk of serious illness and death from COVID-19,^4,11^ worry about access to healthcare services^12,13^ and a heightened fear of HIV-status disclosure during the pandemic.^4,8^ In addition, PLWH noted anxiety surrounding economic hardships associated with the pandemic,^5^ social isolation^3,7^ and increasing stigma around both HIV and SARS-CoV-2 infection.^8,14^ Finally, coping strategies used by PLWH, such as social support groups^15^ may have been compromised by the COVID-19 pandemic.^3,6,16^

However, there is debate as to the extent of pandemic-related adverse mental health outcomes on PLWH, with a minority reporting improvement in mental health^3,7^ and some commenting that resilience learnt from the AIDS pandemic helped them cope with the disruption caused by COVID-19.^8^ There has been little attention paid to the potential psychological implications of long*-*COVID (referring to the continuation of COVID-19 symptoms after acute infection). There is limited information on long-COVID in PLWH, but it is suggested it could be more common compared to the general population,^17^ and often includes psychological burden.^18^ There has also been little focus on the subgroups for which pandemic-related anxiety becomes dysfunctional, which is likely associated with a greater impact on quality of life and different requirements from healthcare providers.^19^

This study aims to further investigate the psychological impact of COVID-19 in PLWH during the pandemic on a UK-based cohort of PLWH recruited to a study exploring immune response to COVID-19 vaccination. An understanding of the impact of the pandemic on the mental health of PLWH will help inform interventions and management in future pandemics, and can help contextualise the current mental health needs of PLWH post-pandemic.

## Methods

### Data collection

Participants were recruited from two inner London HIV outpatient clinics (providing care to approximately 5,000 and 3,000 patients respectively) during the pandemic (from 01/03/2020 to 30/05/2022) as part of an ongoing observational study (Berkshire Research Ethics Committee (REC) 16/SC/0265). The purpose of this study is to characterise immune responses in PLWH with or without a history of COVID-19 disease including the potential psychological impact of the COVID-19 pandemic. Eligible participants (>18 years of age with or without a history of antiretroviral therapy at enrolment) were identified through clinic records and/or clinicians delivering care, and were invited to participate. Self-reported history of exposure or confirmed SARS-CoV-2 infection (measured by SARS-CoV-2 PCR and/or antibody tests) were declared by the participants, who were also asked to provide details on the timing and nature of symptoms. Participants who met any of the following criteria were excluded from the study: unable to give informed consent, current AIDS-defining condition (excluded because the presence of additional co-infections/conditions may confound the immune responses to SARS-CoV-2), pregnant females.^20–22^ Socio-demographic parameters (age, gender, ethnicity, household, education, feeling of economic security (a qualitative outcome used in previous studies that is highly indicative of financial security,^23,24^ history of anxiety/depression, pandemic concerns) were self-reported and HIV/COVID-19-related information (HIV status, HIV viral load, CD4^+^ T cell count, comorbidities, past COVID-19 disease, long COVID symptoms) were collected during routine screening. At the time of recruitment, participants were invited to complete an optional on-site, pen-and-paper questionnaire to assess the potential psychological impact of COVID-19 utilising a brief mental health screening tool, the Coronavirus Anxiety Scale (CAS). This is a unidimensional, validated scale measuring COVID-19-related dysfunctional anxiety,^19^ where a score of ≥9 (out of 20) signifies dysfunctional pandemic-related anxiety. The survey also contained two free-text questions specifically addressing aspects of the pandemic that had an impact on well-being, and coping strategies. See Supplementary Material S1 for a copy of the CAS questionnaire.

### Analysis

As CAS scores were not normally distributed, median CAS scores were used to describe the distribution of scores across the cohort. To analyse the differences in CAS scores for different sociodemographic characteristics, CAS scores were converted into a binary variable summarising whether any anxiety symptoms were present (score of ≥1 or 0) before Chi Squared Tests were performed. This methodology was repeated to assess dysfunctional anxiety, with a validated cut-off of CAS scores ≥9.

## Results

### Demographic characteristics

Our cohort consisted of 115 individuals living with HIV (Table 1). The median age was 51 (range; 22–93), 83.5% (*n* = 96) identified as Male, 58.3% (*n* = 67) were White/Caucasian, and 33.0% (*n* = 38) reported living alone. The majority had undertaken post-secondary school education (82.6%, *n* = 95). Most had well-managed HIV; only 8.7% of the cohort had a current CD4^+^ cell count of <350 cells/mm^3^ (*n* = 10) and 7.0% a viral load >50 copies/mL (*n* = 8). One third (33%, *n* = 38) of participants self-reported pre-pandemic anxiety or depression. Almost two thirds (64.3%, *n* = 74) of the cohort had been exposed to SARS-CoV-2 at the time of assessment.

**Table 1.** Demographic characteristics of our cohort of PLWH.

Sociodemographic characteristic	Number of sample population^1^ (%)
Total	115 (100%)
Sex	
Male	96 (83.5%)
Female	18 (15.7%)
Other	1 (0.9%)
Age	
16-39	16 (13.9%)
40+	85 (73.9%)
Unknown	14 (12.2%)
Ethnicity	
Caucasian/White	67 (58.3%)
Asian	4 (3.5%)
Black African	22 (19.1%)
Other	8 (7.0%)
Living alone	38 (33.0%)
Economic insecurity^2^	28 (24.3%)
No post-secondary education	18 (15.7%)
Number of pandemic concerns^3^	
0/1	36 (31.3%)
2+	78 (67.8%)
Self-reported pre-pandemic anxiety/depression diagnosis	38 (33.0%)
Prior SARS-CoV-2 infection (self-reported/PCR or antibody test)	74 (64.3%)
Experienced long COVID (self-reported)	37 (32.2%)
Hospitalised with COVID-19	8 (7.0%)
Current CD4^+^ count <350 cells/mm^3^	10 (8.7%)
Current HIV viral load ≥50 copies/mL	8 (7.0%)
Report any Comorbidity^4^	62 (53.9%)

### Differences in CAS score for PLWH

All eligible PLWH enrolled over this period completed the optional CAS questionnaire. Overall CAS scores in the cohort were low. 78 participants (67.8%) had a CAS score of 0, thus reporting no COVID-19 related concerns, and 105 (91.3%) reported a score of three or less. The mean and median CAS scores were 1.18 and 0, respectively. Median scores were 0 across all sociodemographic groups specified in Table 1. Supplementary Material S2 shows a histogram of CAS scores across the cohort. The influence of sociodemographic characteristics on CAS score was conducted by investigating which factors were associated with scoring ≥1 (Table 2). Those scoring ≥1 were more likely to report a greater number of specific concerns related to the pandemic (*p = 0.001*) and were more likely to report SARS-CoV-2 exposure (*p = 0.026)*. A higher proportion of individuals who self-reported feelings of economic instability (46.4% versus 27.4%) and who self-reported long COVID (48.6% versus 29.7%) reported CAS scores greater than 0, but this was not statistically significant at the 5% level.

**Table 2.** Proportion of the study population reporting CAS scores greater than or equal to 1, and *p* values from a Chi Squared test for each sociodemographic characteristic.

Sociodemographic characteristic (*n*^5^)	Number with CAS score ≥1 (%^6^)	*p value* ^7^
Total (115)	36 (31.3%)	
Sex		
Male (96)	28 (29.2%)	0.246
Female (18)	7 (38.9%)	
Other (1)	1 (100.0%)	
Age		
16-39 (16)	4 (25.0%)	0.631
40+ (85)	28 (32.9%)	
Ethnicity		
Caucasian/White (67)	20 (29.9%)	0.278
Asian (4)	0 (0.0%)	
Black African (22)	9 (40.9%)	
Other (8)	4 (50.0%)	
Living alone		
Yes (38)	13 (34.2%)	0.571
No (77)	23 (29.9%)	
Economic insecurity^8^		
Yes (28)	13 (46.4%)	0.062
No (85)	23 (27.1%)	
Post-secondary education		
Yes (95)	30 (31.6%)	0.859
No (18)	5 (27.8%)	
Number of pandemic concerns^9^		
0/1 (36)	4 (11.1%)	**0.001**
2+ (78)	32 (41.0%)	
Self-reported pre-pandemic anxiety/depression diagnosis		
Yes (38)	15 (39.5%)	0.341
No (67)	20 (29.9%)	
Prior SARS-CoV-2 infection (self-reported, PCR or antibody test)		
Yes (74)	29 (39.2%)	**0.026**
No (31)	5 (16.1%)	
Experienced long COVID (self-reported)		
Yes (37)	18 (48.6%)	0.096
No (37)	11 (29.7%)	
Hospitalised with COVID-19		
Yes (8)	3 (37.5%)	0.943
No (67)	26 (38.8%)	
CD4^+^ count <350 cells/mm^3^		
Yes (10)	2 (20.0%)	0.602
No (62)	17 (27.4%)	
Viral load ≥50 copies/mL		
Yes (8)	1 (12.5%)	0.229
No (107)	35 (32.7%)	
Report any Comorbidity^10^		
Yes (62)	19 (30.6%)	0.646
No (41)	14 (34.1%)	

### Demographic characteristics of those with dysfunctional pandemic-related anxiety

Five individuals (4.34% of participants) reported dysfunctional anxiety symptomatology (defined by a CAS score ≥9). Of these, three identified as female, all identified as either black or “other minority ethnic” and all had previously had COVID-19, with four self-reporting long COVID. Dysfunctional anxiety symptoms were higher in females compared to males, (16.7% vs 2.1%,*)*; Black African (13.6%) and Other Ethnic Minority (25%) compared to White and Asian participants (0%); and participants reporting feelings of economic instability compared to those not reporting this (10.7% vs 2.4%*)*. The proportion scoring ≥9 was greater in people who reported exposure to SARS-CoV-2 (6.8% versus 0%) and those reporting long-COVID (10.8% versus 2.7%). The data for this analysis is presented in Supplementary Material S3.

### Specific pandemic-related concerns of the population

**Figure 1.**
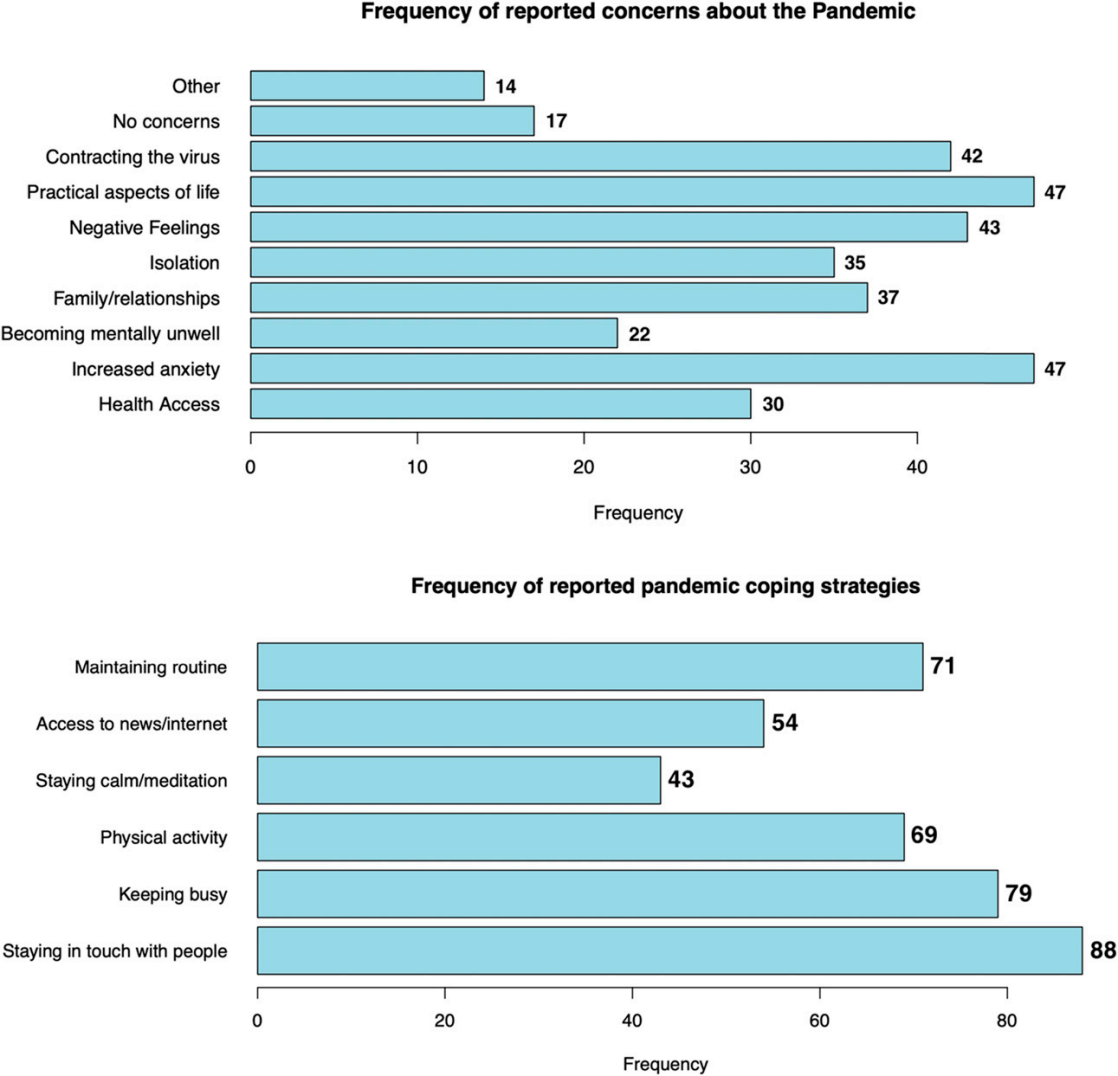
Frequency of reported concerns about the pandemic and coping strategies from our cohort of PLWH.^11^

Two CAS questions quantitatively assessed specific concerns about the pandemic, providing survey respondents with a list of potential pandemic-related experiences (see Supplementary Material S1 for the specific questions in the CAS questionnaire). Figure 1 shows the cohort’s responses to being asked about what aspects they were concerned would impact their mental wellbeing and how individuals were supporting their mental wellbeing during the pandemic.

In addition to this, a quarter (26.1%) of participants completed at least one of two free text answers in the CAS questionnaire (see Supplementary Material S4 for the complete set of responses to the free-text questions). When discussing concerns about the impact of the pandemic on their mental wellbeing, individuals highlighted concerns about their economic situation (“*adjustments to working from home*,” “*risk of employment*”), concerns about the future (“*how return to normal will be*”) and a distrust of decision-makers (“*don’t trust government decision making*”). When asked about coping strategies used during the pandemic, individuals reported social support (“*family weekend lunches, talking to family*,” “*support from friends who’ve also had COVID*”), outdoor activity (“*being outdoors*,” “*gardening*”*)*, and religious practice (“*faith prayer*,” “*study at the church of Scientology*”) as important coping mechanisms. Several concerns and coping behaviours reported may have damaging health implications, both physically (“*increased drinking alcohol*,” “*red wine, cannabis*”) and mentally (“*anxiety*,” “*manage by myself*,” “*lack of motivation, more lonely*”).

## Discussion

Reporting of pandemic-related anxiety in our cohort was low. The mean CAS score for our population was 1.18, which is similar to pandemic-related anxiety levels reported in studies of non-HIV populations using the CAS,^25,26^ and the median score for the cohort and all sociodemographic sub-populations was 0. This could be indicative of strong coping strategies for PLWH within our cohort. This supports previous qualitative findings reporting that previous experience of the HIV/AIDS pandemic and the associated coping strategies that PLWH have developed have helped the population cope with the change and disruption associated with COVID-19.^8,27^ These findings also counter previous studies reporting disproportionate mental health impact on PLWH.^4–10^ Although our findings may be influenced by the demographics of our cohort (predominantly male, educated, economically stable and white), it is important that future work clarifies the psychological impact of pandemics on PLWH, as it will inform future pandemic policy and support.

Our findings found that, despite a small sample size, a higher proportion of women, POC and those self-reporting economic instability reported dysfunctional pandemic-related anxiety. Reasons underlying this trend in a disproportionate impact of the pandemic on women living with HIV could include the burden of unpaid work increasing amongst women,^5^ women experiencing greater work disruption compared to men,^28^ and the pandemic exacerbating pre-existing gender inequalities.^29^ Previous qualitative studies have not found a difference in the mental health impact of the pandemic of ethnic minority PLWH,^3^ but a disproportionate mental health impact of an HIV diagnosis on this population exists independently of the COVID-19 pandemic.^30^ Although our cohort represents a small proportion of PLWH it provides insight into the impact of the pandemic on women and POC living with HIV and should motivate further research into the mental health impact of the pandemic on these sub-populations. Pandemic-related anxiety was not found to be correlated with HIV-related factors, such as CD4 count and viral load, which varies from pre-pandemic mental health research that found PLWH with lower CD4 counts and higher viral load reported worse mental health outcomes.^31,32^ A pre-pandemic diagnosis of depression or anxiety was also not associated with dysfunctional pandemic-related anxiety. There was a statistically significant association between CAS scores greater than 0 and self-reported COVID-19 exposure, and a higher proportion of individuals reporting COVID-19 exposure and long COVID also reported dysfunctional pandemic-related anxiety. The influence of these factors should be further investigated in larger population studies.

The CAS questionnaire included spaces for individuals to provide qualitative answers which provided insight into reasons behind worsening pandemic-related anxiety. Important coping strategies used by PLWH during this period could have been compromised by early pandemic policy advising PLWH to shield and limit social contact,^2^ such as staying in touch with people. This is particularly concerning considering many PLWH are already socially marginalised,^33^ and social isolation has been noted to exacerbate mental health decline in qualitative studies of PLWH.^4,9^ Additionally, some reported coping strategies could have negative health consequences for PLWH, such as increased alcohol consumption and substance misuse. Our findings support qualitative data showing an increase in alcohol consumption^34^ and substance misuse^35^ for PLWH during the pandemic, and this has been found to be associated with depressive symptoms in PLWH.^36^ These findings should be taken into consideration when implementing limitations on PLWH in future pandemics, and as an indicator of potential future clinical needs for PLWH. One approach to supporting pandemic-related anxiety in PLWH is integrating mental health screening and treatment within standard care,^3^ which has been identified as an efficacious approach in supporting the overall mental health of PLWH, particularly for those groups we have identified as greater risk of experiencing dysfunctional pandemic-related anxiety.^37^ Other public health interventions that can be delivered remotely, such as mindfulness interventions, have been effective in improving quality of life in PLWH, and could represent cost- and clinically-effective pandemic-related anxiety management techniques.^3^

Our study is limited by our small sample size of PLWH. Many of our outcomes were also self-reported, including long-COVID and economic situation. The CAS scores only indicated the experience of participants at the moment of data collection and did not represent their anxiety levels throughout the different pandemic phases. Our study population was also predominantly white, male-identifying, educated and economically stable, and likely to be more engaged with accessing the healthcare system. Therefore, our overall findings may not be applicable to the diverse population of PLWH across the UK and internationally.

## Conclusion

In a UK-based cohort of PLWH, self-reported pandemic-related anxiety was low, potentially indicating the resilience of the population and coping strategies carrying over from the HIV/AIDS pandemic. PLWH who had had COVID-19 disease reported significantly worse pandemic-related anxiety than those who had not had COVID-19. The small proportion of our cohort who reported dysfunctional pandemic-related anxiety were predominantly female-identifying, Black African/Other Ethnic Minority and reported economic instability. Future research should investigate these potential subpopulations of PLWH most at risk of dysfunctional pandemic-related anxiety, and clinicians should work to address the mental health needs of this group.

## Supplemental Material

Investigation into the psychological impact of the COVID-19 pandemic for people living with HIV Click here for additional data file. Supplemental material for Investigation into the psychological impact of the COVID-19 pandemic for people living with HIV by Sze Wing Karina Lo, Luke Muschialli, Thomas Fernandez, Colette Smith, Dimitra Peppa and Fiona Burns in International Journal of STD & AIDS Journal.

Investigation into the psychological impact of the COVID-19 pandemic for people living with HIV Click here for additional data file. Supplemental material for Investigation into the psychological impact of the COVID-19 pandemic for people living with HIV by Sze Wing Karina Lo, Luke Muschialli, Thomas Fernandez, Colette Smith, Dimitra Peppa and Fiona Burns in International Journal of STD & AIDS Journal.

Investigation into the psychological impact of the COVID-19 pandemic for people living with HIV Click here for additional data file. Supplemental material for Investigation into the psychological impact of the COVID-19 pandemic for people living with HIV by Sze Wing Karina Lo, Luke Muschialli, Thomas Fernandez, Colette Smith, Dimitra Peppa and Fiona Burns in International Journal of STD & AIDS Journal.

Investigation into the psychological impact of the COVID-19 pandemic for people living with HIV Click here for additional data file. Supplemental material for Investigation into the psychological impact of the COVID-19 pandemic for people living with HIV by Sze Wing Karina Lo, Luke Muschialli, Thomas Fernandez, Colette Smith, Dimitra Peppa and Fiona Burns in International Journal of STD & AIDS Journal.

## Notes

1 Not all percentages may add to 100% in the cohort overall due to the small amount of missing data where participants did not answer the question, or questions not being applicable.

2 Defined by those who self-reported not always having enough money to satisfy basic needs.

3 Pandemic concerns were measured by individuals reporting whether they had experienced one of a list of pandemic related concerns provided by the CAS questionnaire. These were any of: access to healthcare, increased anxiety, becoming mentally unwell, family and relationships, isolation, negative feelings, practical aspects of life, concerns about yourself/your family contracting the virus and other (a free text box for individuals to express specific concerns).

4 Comorbidities were self-reported from a list of comorbidities provided, which were: hypertension, cardiovascular disease (CVD), ischaemic heart disease, chronic obstructive pulmonary disease (COPD), asthma.

5 Values may not add to the total value due to a small number of missing data.

6 Percentages are calculated as the proportion of the subset of the total study population reporting a CAS score ≥ 1. For example, 29.2% of the 92 men in the study population reported a CAS score ≥ 1.

7 Bold values represent statistical significance (*p <* 0.05).

8 Defined by those who self-reported not always having enough to satisfy basic needs.

9 Pandemic concerns were measured by individuals reporting whether they had experienced one of a list of pandemic related concerns provided by the CAS questionnaire. These were: access to healthcare, increased anxiety, becoming mentally unwell, family and relationships, isolation, negative feelings, practical aspects of life, concerns about yourself/your family contracting the virus and other (a free text box for individuals to express specific concerns).

10 Comorbidities were self-reported from a list of comorbidities provided, which were: hypertension, cardiovascular disease (CVD), ischaemic heart disease, chronic obstructive pulmonary disease (COPD), asthma.

11 Labels on the *y*-axis are abbreviated forms of the options given to respondents on the CAS questionnaire. The full titles of the options given to participants can be found in Supplementary Material 1.
