# Supplementary material for: Investigation into the psychological impact of the COVID-19 pandemic for people living with HIV
Source: Int J STD AIDS. 2023 Jun 3;34(11):777–84. doi: 10.1177/09564624231179275 (PMC10240304; doi:10.1177/09564624231179275)
Supplement: Investigation into the psychological impact of the COVID-19 pandemic for people living with HIV [file sj-pdf-1-std-10.1177_09564624231179275.pdf]

COVID-19 Study Participant Questionnaire V1 29042020

Participant code :

Study no: ______________________

<u>Optional Questionnaire</u>

<u>Which of the following living arrangements best describes your household?</u>

Live alone ☐
Live with partner/spouse ☐
Live with partner/spouse/children ☐
Live with parents or other related adults ☐
Live with other unrelated adults ☐
Other (please specify):______________________

<u>What is your highest level of education:</u>

No formal education ☐
High-School ☐
Vocational training ☐
University ☐
Other (please specify): ______________________

<u>Do you have enough money to cover your basic needs?</u>

Always ☐ Mostly ☐ Sometimes ☐ No ☐

Thinking about the current coronavirus (COVID-19) pandemic, what if any concerns do you have about the impact on your mental wellbeing? Please tick any that apply.

1. Access to health support and services ☐
2. Increased anxiety ☐
3. Becoming mentally unwell ☐
4. Family and relationships ☐
5. Isolation ☐
6. Negative feelings ☐
7. Practical aspects of life (i.e finances, employment, impact on business) ☐
8. Concern about yourself or your family contracting the virus ☐
9. No concerns ☐
10. Other please specify:______________________

We want to understand what people are doing to support their mental wellbeing during the COVID-19 pandemic. What if anything has been helping your mental wellbeing at this time?

1. Staying in touch with friends and family ☐
2. Keeping busy ☐
3. Physical activity ☐
4. Staying calm/meditation ☐
5. Access to Information (i.e News, internet) ☐
6. Maintaining routine ☐

7. Other please specify:____

Prior to the COVID-19 pandemic did you suffer with anxiety or depression?
Yes ☐ No ☐ Prefer not to say ☐

Please read each statement and circle what applies to you. Please give one answer per column.

**CAS**						
	**How often have you experienced the following over the last 2 weeks?**	**Not at all**	**Rare, less than a day or two**	**Several days**	**More than seven days**	**Nearly every day over the last 2 weeks**
1	I felt dizzy, lightheaded or faint, when I read or listened to the news about the coronavirus	0	1	2	3	4
2	I had trouble falling or staying asleep because I was thinking about the coronavirus	0	1	2	3	4
3	I felt paralysed, or frozen when I thought about or I was exposed to information about the coronavirus	0	1	2	3	4
4	I lost interest in eating when I thought about or I was exposed to information about the coronavirus	0	1	2	3	4
5	I felt nauseous or had stomach problems when I thought about or I was exposed to information about the coronavirus	0	1	2	3	4
	Column Totals					
					Total Score:	
