# Supplementary material for: Investigation into the psychological impact of the COVID-19 pandemic for people living with HIV
Source: Int J STD AIDS. 2023 Jun 3;34(11):777–84. doi: 10.1177/09564624231179275 (PMC10240304; doi:10.1177/09564624231179275)

*Supplementary Material 2: Histogram of the distribution of total Coronavirus Anxiety Scale (CAS) scores*

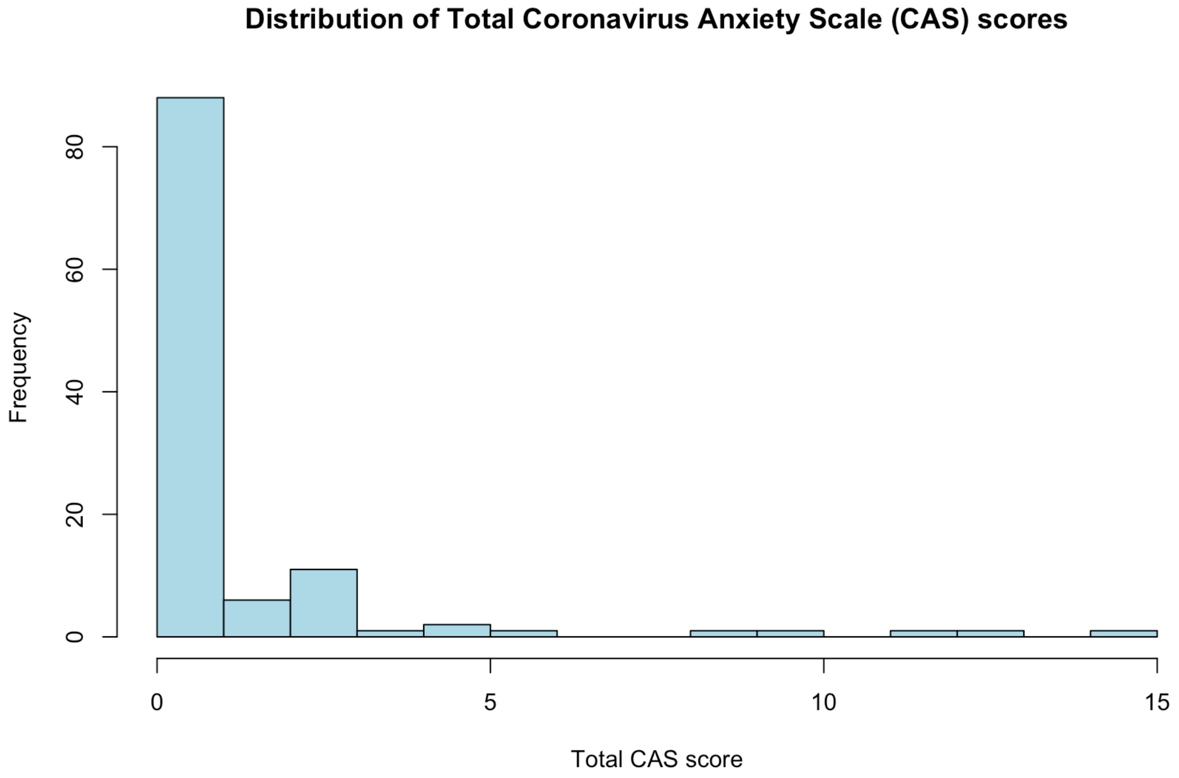

Supplement: Investigation into the psychological impact of the COVID-19 pandemic for people living with HIV [file sj-pdf-2-std-10.1177_09564624231179275.pdf]
