# Supplementary material for: Investigation into the psychological impact of the COVID-19 pandemic for people living with HIV
Source: Int J STD AIDS. 2023 Jun 3;34(11):777–84. doi: 10.1177/09564624231179275 (PMC10240304; doi:10.1177/09564624231179275)
Supplement: Investigation into the psychological impact of the COVID-19 pandemic for people living with HIV [file sj-pdf-3-std-10.1177_09564624231179275.pdf]

Supplementary Material 3: Analysis on the influence of sociodemographic characteristics on CAS score (dysfunctional anxiety - ≥ 9)

Sociodemographic characteristic	CAS score ≥ 9 (%[1])	*p value*[2]
Sex		
Male	2.1	**0.021**
Female	16.7	
Other	0	
Age		
16-39	0	0.335
40+	5.9	
Ethnicity		
Caucasian/White	0	**0.003**
Asian	0	
Black African	13.6	
Other	25	
Living alone		
Yes	5.4	0.713
No	3.9	
Economic insecurity[3]		
Yes	10.7	0.064
No	2.4	
Post-secondary education		
Yes	4.2	0.759
No	5.9	
Number of pandemic concerns[4]		
0/1	0	0.118
2+	6.5	
Self-reported pre-pandemic anxiety/depression diagnosis		
Yes	7.9	0.264
No	3.0	
Prior COVID-19 Infection		

[1] Percentages are calculated as the proportion of the subset of the total study population reporting a CAS score ≥ 1. For example, 29.2% of the 92 men in the study population reported a CAS score ≥ 1.
[2] The Chi Squared test was used to determine statistical significance
[3] Defined by those who self-reported not always having enough to satisfy basic needs
[4] Pandemic concerns were measured by individuals reporting whether they had experienced one of a list of pandemic related concerns provided by the CAS questionnaire. These were: access to healthcare, increased anxiety, becoming mentally unwell, family and relationships, isolation, negative feelings, practical aspects of life, concerns about yourself/your family contracting the virus and other (a free text box for individuals to express specific concerns).

(self-reported)		
Yes	6.8	0.145
No	0	
Experienced long COVID (self-reported)		
Yes	10.8	0.165
No	2.7	
Hospitalised with COVID-19		
Yes	0	0.424
No	7.5	
CD4+ Count < 350		
Yes	0	0.561
No	3.3	
Viral load ≥ 50		
Yes	0	0.530
No	4.7	
Comorbidities[5]		
Yes	4.8	0.971
No	5.0	

[5] Comorbidities were self-reported from a list of comorbidities provided, which were: hypertension, cardiovascular disease (CVD), ischaemic heart disease, chronic obstructive pulmonary disease (COPD), asthma.
