# Supplementary material for: Investigation into the psychological impact of the COVID-19 pandemic for people living with HIV
Source: Int J STD AIDS. 2023 Jun 3;34(11):777–84. doi: 10.1177/09564624231179275 (PMC10240304; doi:10.1177/09564624231179275)
Supplement: Investigation into the psychological impact of the COVID-19 pandemic for people living with HIV [file sj-pdf-4-std-10.1177_09564624231179275.pdf]

Supplementary Material 4: Complete Free-Text Responses from Study Population

1. **Thinking about the current coronavirus (COVID-19) pandemic, what, if any, concerns do you have about the impact on your mental wellbeing? Please tick any that apply.**

- Difficulty of travelling (parents living abroad)
- Don’t trust government decision making and management
- Increased tiredness and low mood and stress
- Lack of motivation, more lonely
- Anxiety
- Felt anxiety at start of pandemic especially re. providing care for patients
- Family abroad and partner abroad
- Increased drinking alcohol, went to AA
- Newfill face treatments stopped. Getting facial wasting – depression
- Worries about the future
- None, I’m A-Ok
- Risk to employment
- Starting my own business
- It hasn’t adversely affected me, after having already experienced depression and anxiety
- How return to the new normal will be
- Lost of income
- Adjustements to working from home
- Inability to travel

2. **We want to understand what people are doing to support their mental wellbeing during the COVID-19 pandemic. What, if anything, has been helping your mental wellbeing at this time?**

- Being outdoors
- Reading, music
- Hobbies – rediscovering old ones. Support from friends who’ve also had COVID
- Gardening
- Going to work, family weekend lunches, talking to family
- Faith prayer
- Manage by myself
- Watching less TV, shopping routines
- IAPT services, sertraline
- Red wine, cannabis
- Study at the church of scientology
- Prayer,
- Netflix, cats, going to work

- Support groups, gym, park, eating well
- Sat it out
- My dog
- Improvement projects
- Dog
- Focusing on selflove
- Counselling helped
- Online free courses
